# Identification and Expression Analysis of the Potato (*Solanum tuberosum* L.) stu-miR482 Family Under Exogenous 24-Epibrassinolide Treatments and Alkaline Salt Stress

**DOI:** 10.3390/plants15121856

**Published:** 2026-06-15

**Authors:** Jing Wang, Yong Wang, Yuan Lu, Xingxing Wang, Yunyun Du, Weina Zhang, Yichen Kang, Shuhao Qin

**Affiliations:** College of Horticulture, Gansu Agricultural University, Lanzhou 730070, China; wj301202@163.com (J.W.); wangy5516@163.com (Y.W.); 19909468161@163.com (Y.L.); wangxingxing1107@163.com (X.W.); 18293955267@163.com (Y.D.); zhangwn@gsau.edu.cn (W.Z.); kangyc@gsau.edu.cn (Y.K.)

**Keywords:** 24-epibrassinoside, alkaline salt stress, expression analysis, miR482, potato

## Abstract

Potato (*Solanum tuberosum* L.) is the world’s fourth-largest staple crop. Alkaline salt stress is a major abiotic stress factor that severely limits the growth, yield, and quality of potatoes; however, little is known about the molecular basis of potatoes’ response to alkaline salt stress or the stress-alleviation mechanism mediated by 24-epibrassinoside. In this study, we conducted a genome-wide identification of the potato miR482 family and analyzed its response patterns under alkaline salt stress and 24-epibrassinoside-mediated stress relief. We identified a total of 9 mature stu-miR482 sequences and 5 precursor sequences; all precursors form typical stable hairpin structures and exhibit high evolutionary conservation among *Solanaceae* plants. Promoter analysis revealed multiple cis-acting elements in the promoter region associated with light signaling, plant hormones, and stress signaling. A total of 64 potential target genes were predicted, encompassing transcription factors, disease resistance, and signal transduction-related genes, forming a complex regulatory network. Phenotypic analysis confirmed that EBR significantly alleviates the growth inhibition in potatoes induced by alkaline salt stress. qRT-PCR analysis indicated that stu-miR482a-5p is the primary stress-responsive member in leaves; stu-miR482d-3p/5p exhibited the strongest regulatory response to EBR in roots; in potato stolons, all members of the miR482 family were significantly upregulated under alkaline salt stress, with stu-miR482d-5p showing extremely significant upregulation across all treatment groups. In summary, this study represents the first systematic characterization of the potato miR482 family, revealing its tissue differential functions in alkaline salt stress and EBR-mediated stress relief.

## 1. Introduction

The potato (*Solanum tuberosum* L.) is the world’s fourth-largest food crop, surpassed only by rice, wheat, and corn, and is widely cultivated and consumed worldwide [1]. However, as soil salinization continues to worsen globally, salt and alkali stress have become one of the primary abiotic stress factors limiting potato growth and yield [2]. In the arid and semi-arid regions of northwest China, soil salinization has intensified due to a variety of factors, including scarce precipitation, excessive application of chemical fertilizers and pesticides, continuous cropping obstacle and land overexploitation [3]. Alkaline soils are typically characterized by high pH (>8.5), high exchangeable sodium content, poor fertility, poor physical properties, and extremely low water permeability. Plants growing in such soils are not only affected by sodium toxicity but also suffer from CO_3_^2−^/HCO_3_^−^ stress and high pH stress [4]. High pH levels in saline-alkali soils can impair plant growth and disrupt ionic balance; therefore, the risk posed by alkaline salt stress may be greater than that posed by neutral salt (NaCl) stress [5,6]. Currently, mitigating the damage to potatoes caused by alkaline salt stress is a top priority in potato breeding. Brassinosteroids (BRs) are a class of steroid-type plant hormones that play a key regulatory role in numerous physiological processes related to plant growth, development, and stress responses [7]. The exogenous application of brassinosteroids promotes the growth and yield of many plants by regulating seed germination, protein content, seedling growth, proline content, lipid peroxidation, photosynthetic capacity, and water relations [8,9,10]. In terms of responses to abiotic stress, BRs can enhance plants’ overall stress tolerance by increasing the activity of antioxidant enzymes, optimizing the antioxidant defense system, and maintaining intracellular homeostasis [11]. This property enables it to effectively help plants withstand various abiotic stresses, including high and low temperatures, drought, salinity, pesticide residues, and heavy metal contamination [12,13,14,15,16,17]. Previous studies have shown that exogenous application of 24-epibrassinolide (EBR) to potato plants can significantly enhance their tolerance to alkaline salt stress by improving the function of the plant’s antioxidant system and photosynthetic physiological characteristics [18].

During long-term evolution, plants have developed complex stress-resistant regulatory networks. Among these networks, microRNAs (miRNAs)—a class of small non-coding RNAs approximately 20–24 nt in length—guide the targeted cleavage of target mRNAs or repress their translation via complementary base pairing, and play a core regulatory role in plant growth and development, hormone signal transduction, and stress responses [19,20]. Many miRNAs have been shown to play important roles in plant stress responses. Several plant miRNAs, such as miR156, miR160, miR172, miR393, miR166, and miR169, are involved in responses to salt, drought, water deficiency, and salinity stress [20,21,22,23]. In addition, miRNAs regulate plant growth and development, hormone responses, and stress responses through their target genes [24,25,26]. The miR482 family, one of the plant-specific miRNA families, primarily targets nucleotide-binding site-leucine-rich repeat (NBS-LRR) disease resistance genes and was initially found to be involved in plant immune responses against pathogens [27]. As research progresses, growing evidence suggests that members of the miR482 family may participate in responses to abiotic stresses through their target genes. For example, Eca-miR482f in *Eucalyptus camaldulensis* contributes to the plant’s response to cold stress by targeting the *EcaSIZ1* gene, a key component of the ICE1–CBFs–CORs cold signaling pathway [28]. A similar pattern of target gene expansion is observed in soybean: under salt stress, the expression of gma-miR482bd-5p is significantly reduced, while its predicted targets, *HEC1* and *BAK1*, are correspondingly upregulated, indicating that miR482 participates in the plant stress response by negatively regulating these target genes [29]. In addition, the expression of miR482 family members is also involved in the regulation of hormonal signaling; hormones such as gibberellin (GA), methyl jasmonate (MeJA), and auxin α-naphthaleneacetic acid (NAA) can all influence their expression levels [30,31,32]. It is worth noting that EBR can mediate miRNA expression, specifically regulating the transcription or translation of downstream functional genes, thereby influencing plant growth and development. For example, in Arabidopsis, EBR significantly upregulates the expression of miR395a, thereby specifically suppressing the expression of the multifunctional protein gene *GUN5* and its downstream signaling pathways, ultimately achieving precise regulation of *Arabidopsis* seedling morphogenesis and root growth [33].

The miR482 family is a plant-specific non-coding RNA that plays a key regulatory role in abiotic stress responses [27]. However, the expression patterns of the potato miR-482 family under alkaline salt stress and EBR treatment remain unclear. We hypothesize that exogenous EBR alleviates alkaline salt stress by regulating specific members of the stu-miR482 family. Therefore, this study aims to identify members of the stu-miR482 family, analyze their molecular characteristics and phylogenetic conservation, predict target genes and regulatory networks, and investigate their expression patterns under alkaline salt stress and EBR treatment. These efforts will elucidate regulatory roles in stress responses of the stu-miR482 family and provide critical miRNA resources and theoretical foundations for the molecular breeding of salt- and alkali-tolerant potatoes.

## 2. Result

### 2.1. Identification and Evolutionary Analysis of Potato stu-miR482s

**Figure 1:**
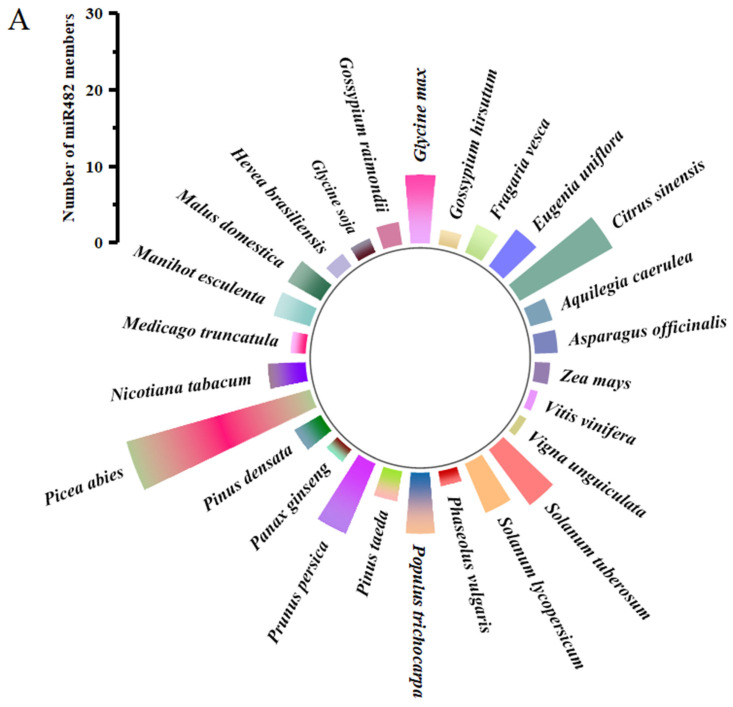

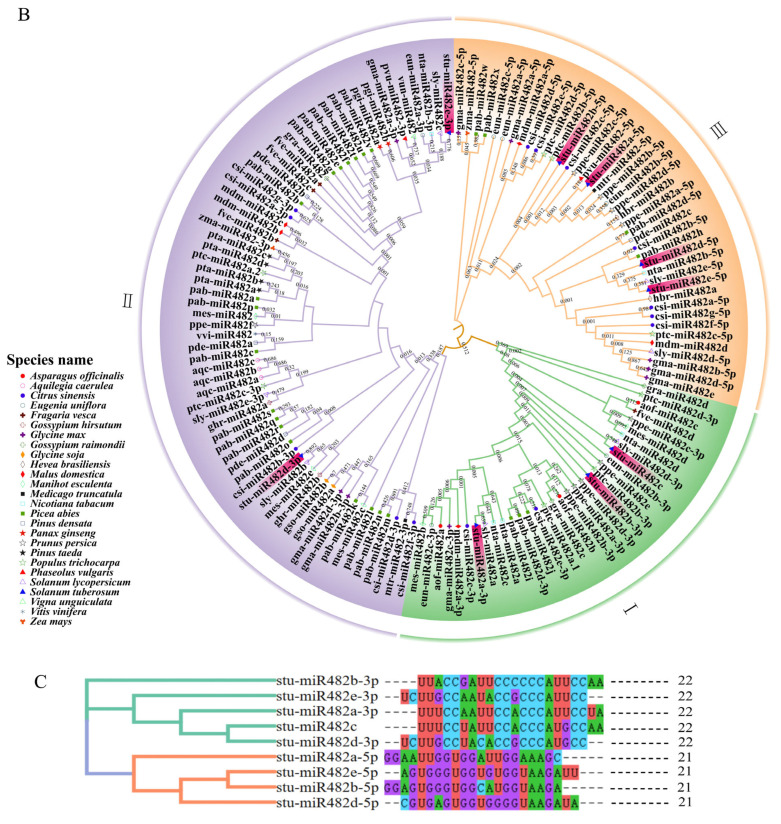
Numeric distribution, phylogeny and multiple sequence alignment of plant miR482 gene family. (**A**) Numeric distribution of miR482s in 26 plant species. bar length represents the number of miR482. (**B**) Phylogenetic tree of plants miR482 mature sequences. Symbols of different colors represent different species. The ones highlighted in red are potato. Different species are marked with different shapes and colors. (**C**) Clustering and multiple sequence comparison of miR482 family members of potato plant.

To investigate the distribution of the miR482 family in plants, a total of 140 mature miR482 sequences from 26 species were downloaded from miRBase, and 9 mature sequences belonging to the potato stu-miR482 family were identified. The results showed that miR482 was most abundant in *Picea abies*, with 25 members, followed by *Citrus sinensis*, with 14 members (Figure 1A). To further investigate the evolutionary relationships of miR482 in plants, a phylogenetic tree was constructed using 140 mature miR482 sequences from 26 plant species, including *Solanum tuberosum* (9), *Nicotiana benthamiana* (5), *Solanum lycopersicum* (7), *Zea may* (2), *Vitis vinifera* (1), *Prunus persica* (10), *Glycine max* (9), and *Picea abies* (25), among 26 plant species, to construct a phylogenetic tree (Figure 1B).

The results showed that the phylogenetic tree was divided into three branches. All stu-miR482s derived from the 5′ end of the precursor belong to clade III. Clade I consists of stu-miR482a-3p, stu-miR482b-3p, and stu-miR482c, all derived from the 3′ end of the precursor. Clade III consists of stu-miR482d-3p and stu-miR482e-3p. Further comparison of the mature sequences of miR482 in potato revealed that stu-miR482 is primarily divided into two types: 5′ end sequences and 3′ end sequences, with the 5′end sequences all being 22 nt in length and the 3′ end sequences being 21 nt in length (Figure 1C). miR482 members grouped, such as stu-miR482a-3p, stu-miR482b-3p, stu-miR482c, stu-miR482d-3p, and stu-miR482e-3p, exhibit higher sequence similarity and greater nucleotide conservation.

### 2.2. Sequence Alignment, Secondary Structure, and Phylogenetic Analysis of Potato stu-pre-miR482s

**Figure 2:**
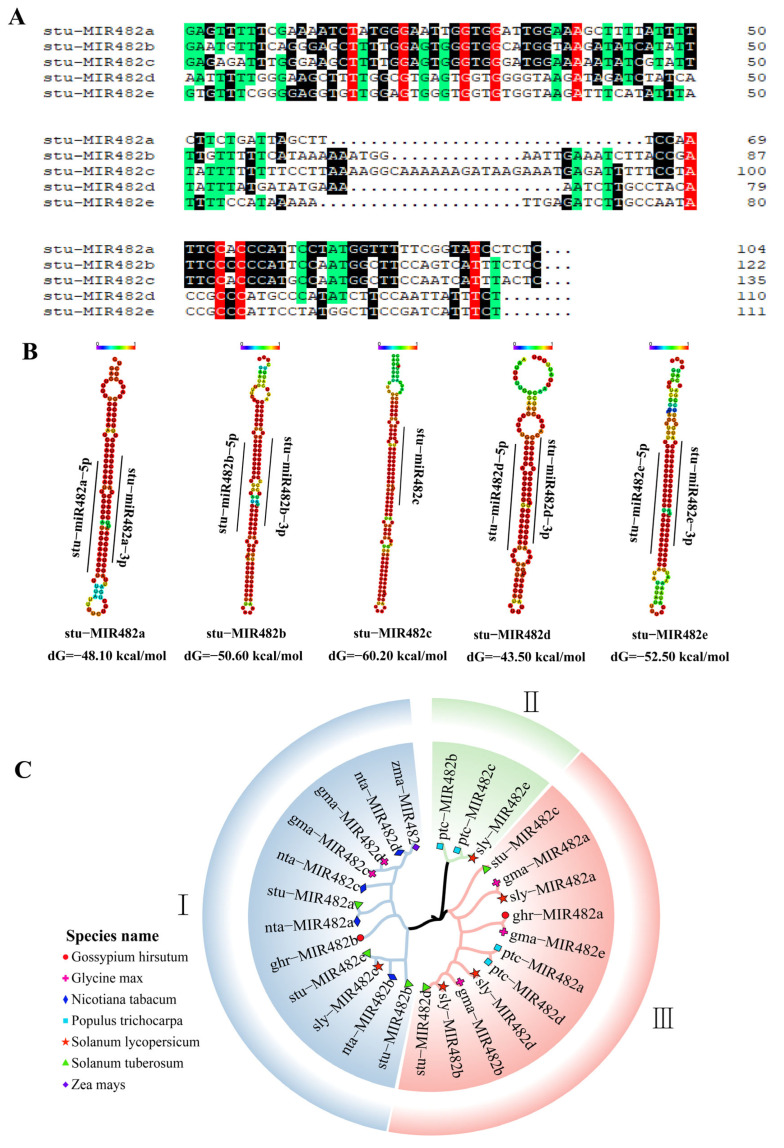
Sequence alignment, secondary structure, and phylogenetic analysis of potato pre-miR482s. (**A**) Sequence comparison of potato plant pre-miR482s, Different highlight colors indicate different sequence homology degree, Red ≥ 100%; Green ≥ 75%; Black ≥ 50%. (**B**) Phylogenetic tree of plants MIR482 sequences, ghr. *Gossypium hirsutum*; gma. *Glycine max*; nta. *Nicotiana tabacum*; ptc. *Populus trichocarpa*; sly. *Solanum lycopersicum*; stu. *Solanum tuberosum*; zma. *Zea mays*. (**C**) Stem-loop structures of Stu-MIR482s in Solanum tuberosum. The mature miRNA sequences are marked with a black line, colors from blue to red indicate the probability of base pairing from 0 to 1.

Based on published small RNA sequencing results, five precursor sequences of the miR482 family were identified in potato. The identified stu-pre-miR482s are all within 300 nt, falling within the normal range for plant miRNA precursor lengths, and their sequences are conserved at the 5′ and 3′ ends (Figure 2A). To determine the structural stability of the miRNAs, the secondary structures of the five precursor sequences of the potato miR482 family members were predicted and analyzed (Figure 2B). It was found that each potato miR482 precursor sequence forms a hairpin structure; the minimum free energy of the miR482 secondary structure is close to the optimal thermodynamic stability value for miRNA stem-loop structures (ΔG = −45.98 kcal/mol), indicating that the structures of all members of the potato miR482 family are relatively stable. Furthermore, by marking the positions of the mature sequences on the secondary structure of the precursors, it was found that the secondary structure of stu-miR482s is highly stable in the region (arm) of the mature miRNA sequence, while the remaining sequence (stem) is relatively unstable.

To further investigate the evolutionary relationships among the five stu-pre-miR482 sequences, the MEGA software was used to analyze the evolutionary relationships of the pre-miR482s sequences in *Gossypium hirsutum* (2), *Glycine max* (5), *Nicotiana benthamiana* (4), *Populus trichocarpa* (4), *Solanum lycopersicum* (5), *Solanum tuberosum* (5), and *Zea mays* (5). The pre-miR482 sequences are primarily divided into three branches. Specifically, one ghr-MIR482, two gma-MIR482s, four nta-MIR482s, one sly-MIR482, three stu-MIR482s, and one zma-MIR482 form a major branch. The second branch includes 2 ptc-MIR482s and 1 sly-MIR482. Members comprising 1 ghr-MIR482, 3 gma-MIR482s, 2 ptc-MIR482s, 3 sly-MIR482s, and 2 stu-MIR482s are grouped into the third branch (Figure 2C). These results indicate that the high conservation of miR482 among *Solanaceae* species reflects their close evolutionary relationships. Furthermore, miR482 from potato clusters is conserved with that of interspecific species such as soybeans and *Populus trichocarpa*, indicating that miR482 exhibits cross-family conservation during evolution and likely performs fundamental biological functions.

### 2.3. Analysis of Transcriptional Regulatory Elements in the stu-miR482s Promoter Region

**Figure 3:**
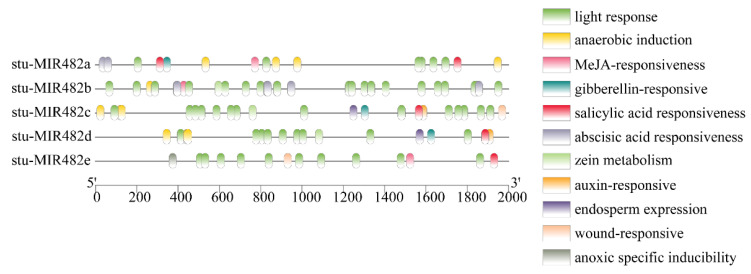
Promoter analysis of stu-MIR482s genes in potato. The gray lines indicate the length of stu-MIR482s promoter sequences. Different colored boxes on the right represent cis-elements with different functions.

Transcription initiation is central to gene expression, with the promoter serving as a key region; elucidating the cis-acting elements of miRNA promoters can clarify their transcriptional activation mechanisms and regulatory functions [34]. To elucidate the biological function of stu-MIR482s, the 2 kb upstream promoter region was analyzed using the PlantCARE platform (Figure 3), revealing 12 major cis-regulatory elements classified into four categories: phytohormone response, abiotic stress response, tissue/metabolism-related, and light response. The characteristics of the elements within each family vary: stu-MIR482a is enriched with light-responsive, anaerobic-inducible, and hormone-responsive elements; stu-MIR482b primarily contains light-responsive, abscisic acid, and zymolytic protein metabolism-related elements; stu-MIR482c is centered on light-responsive elements and also contains salicylic acid, gibberellin-responsive elements, and unique auxin-responsive elements; stu-MIR482d shares similar characteristics with stu-MIR482c but additionally contains anaerobic-inducible elements; stu-MIR482e is centered on light-responsive elements and also contains salicylic acid, methyl jasmonate, and other responsive elements. The identified diverse cis-acting elements imply that stu-MIR482 members may have potential responses to various hormones and stress signals, and could be regulated by multiple regulatory pathways at the transcriptional level.

### 2.4. Analysis of Predicted miR482 Target Genes in Potato

**Figure 4:**
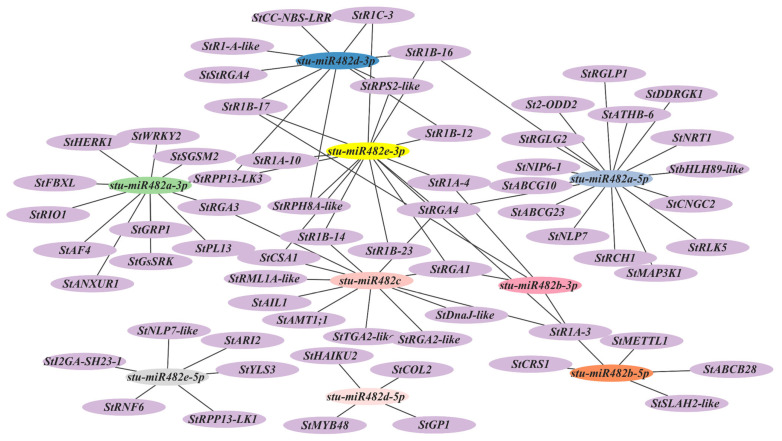
Regulatory network of the stu-miR482s and targets in the potato plant. The different colors of the ellipses in the center represent different mature forms of miR482, while the purple ellipse indicates the target gene.

To further investigate the function of miR482 in potatoes, putative targets of miR482 were predicted using psRNATarget (2017 Update) and homologous sequences were identified by NCBI, resulting in the prediction of 64 potential target genes. A regulatory network diagram was constructed between the above target genes and stu-miR482s (Figure 4). The results showed that members of the potato miR482 family exhibit a broad spectrum of target gene regulation. For example, stu-miR482a-3p and stu-miR482a-5p exhibit targeting associations with a large number of mRNAs, including transcription factor genes (e.g., *StWRKY2*, *StMYB48*), disease resistance-associated genes (e.g., *StCC-NBS-LRR*, *StRGA3*, *StRGA4*), signal transduction-associated genes (e.g., *StCNGC2*, *StMAP3K1*), and other functional genes (e.g., *StYLS3*, *StGP1*). This suggests that the miR482 family may play a key regulatory role in multiple biological processes in potatoes, including growth and development, immune defense, and signal transduction.

### 2.5. Analysis of miR482 Family Member Expression in Potato Aboveground Tissues in Response to Alkaline Salt Stress and EBR

**Figure 5:**
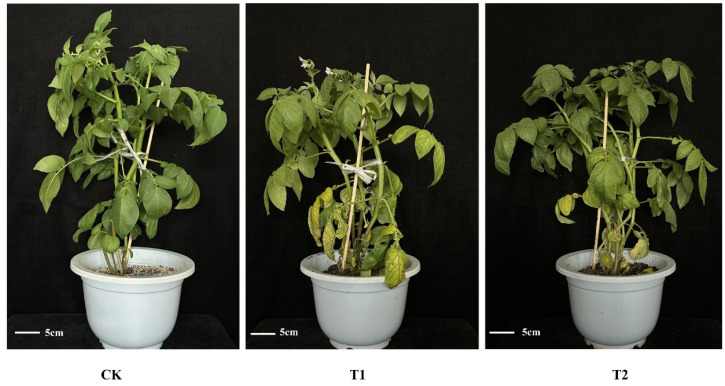
Phenotypes of Atlantic potato potted seedlings under different treatment conditions. CK: Normal growth conditions; T1: 200 mmol·L^−1^ NaHCO_3_; T2: 200 mmol·L^−1^ NaHCO_3_ + 0.1 μmol·L^−1^ EBR.

**Figure 6:**
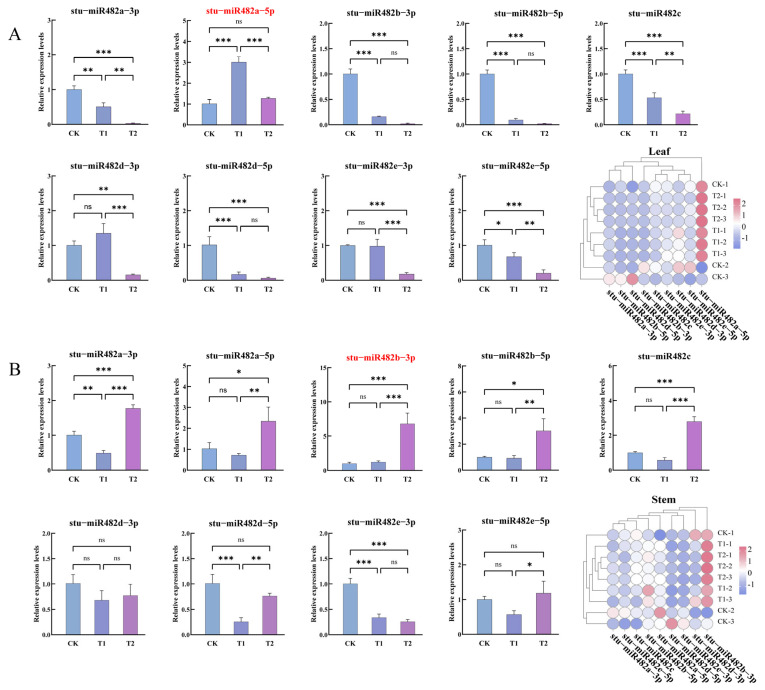
Expression of stu-miR482s in potato aboveground tissues under alkaline salt stress and EBR relief. CK: Normal growth conditions; T1: 200 mmol·L^−1^ NaHCO_3_; T2: 200 mmol·L^−1^ NaHCO_3_ + 0.1 μmol·L^−1^ EBR. (**A**) Leaf tissue expression patterns. (**B**) Steam tissue expression patterns. (ns *p* > 0.05, * *p* < 0.05, ** *p* < 0.01, *** *p* < 0.001).

Through experiments with potted potato plants, we found that alkaline salt stress significantly inhibits potato growth and causes leaf damage, whereas exogenous EBR effectively alleviates this stress, leading to marked improvements in plant growth and leaf condition (Figure 5). Therefore, we used qRT-PCR to examine the expression patterns of stu-miR482s in potato leaves and stems under alkaline salt stress and EBR relief (Figure 6). The results indicate that alkaline salt stress and exogenous EBR significantly regulate the expression of miR482 family members in potato leaves (Figure 6A). Compared with the CK group, the expression levels of stu-miR482a-5p and stu-miR482d-3p were significantly elevated under T1 treatment, while the expression levels of the remaining members were significantly downregulated compared to the CK group. Following T2 treatment, the expression level of stu-miR482a-5p was significantly downregulated compared to T1, but remained 1.27-fold higher than that of the CK group; stu-miR482a-3p, stu-miR482c, stu-miR482d-3p, stu-miR482e-3p, and stu-miR482e-5p were significantly downregulated compared to T1; however, there was no significant difference in the expression levels of stu-miR482b-3p, stu-miR482b-5p, and stu-miR482d-5p between the T2 and T1 groups. Heatmap clustering analysis indicated that stu-miR482a-5p was lowly expressed in CK and significantly upregulated in T1 and T2, making it a core differentially expressed member in response to the treatment.

In potato stems (Figure 6B), compared with CK, the expression levels of stu-miR482a-3p, stu-miR482d-5p, and stu-miR482e-3p were extremely significantly downregulated under alkaline salt stress, while the expression of stu-miR482a-5p, stu-miR482b-3p, and others showed no significant changes. Following T2 treatment, the expression of most miR482 members showed a clear upregulation trend: the expression levels of stu-miR482a-3p, stu-miR482a-5p, stu-miR482b-3p, stu-miR482b-5p, and stu-miR482c were significantly upregulated compared to the CK and T1 groups; There was no significant difference in the expression of stu-miR482d-3p and stu-miR482e-3p between the T2 and T1 groups; heatmap cluster analysis indicated that the expression of miR482 family members in potato stem tissue was significantly regulated by treatment conditions, and different members within the family exhibited distinct expression response patterns. Among these, stu-miR482b-3p, stu-miR482d-3p, and stu-miR482e-3p were identified as the core differentially expressed miRNAs in stem tissue in response to the treatment.

### 2.6. Analysis of miR482 Family Member Expression in Potato Underground Tissues in Response to Alkaline Salt Stress and EBR

**Figure 7:**
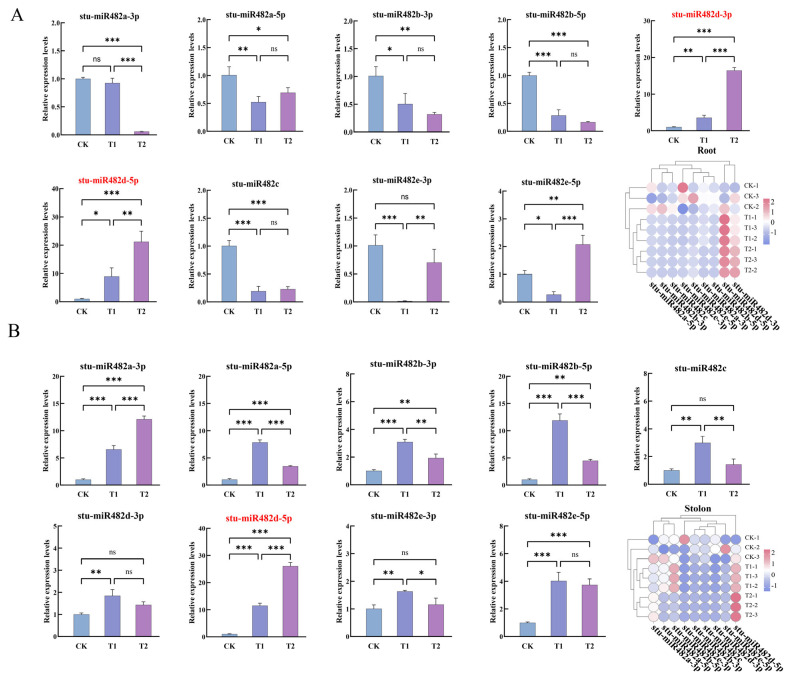
Expression of stu-miR482s in potato underground parts under alkaline salt stress and EBR relief. CK: Normal growth conditions; T1: 200 mmol·L^−1^ NaHCO_3_; T2: 200 mmol·L^−1^ NaHCO_3_ + 0.1 μmol·L^−1^ EBR. (**A**) Root tissue expression patterns. (**B**) Stolon tissue expression patterns. (ns *p* > 0.05, * *p* < 0.05, ** *p* < 0.01, *** *p* < 0.001).

To analyze the response of potato miR482 family members to alkaline salt stress and exogenous 24-epibrassinoside in their underground parts, RT-qPCR was used to detect the expression patterns of stu-miR482s in potato roots and stolons under alkaline salt stress and EBR relief (Figure 7). The results indicate that alkaline salt stress and exogenous 24-epibrassinoside significantly regulate the expression of miR482 family members in potato roots (Figure 7A). Compared with CK, after T1 treatment, the expression of most members, such as stu-miR482a-5p, stu-miR482c, and stu-miR482e-3p, was significantly decreased. In contrast, following T2 treatment, the expression levels of stu-miR482d-3p, stu-miR482d-5p, stu-miR482e-3p, and stu-miR482e-5p were all significantly increased compared to the T1 group; moreover, the expression of stu-miR482d-3p and stu-miR482d-5p even exceeded the levels observed in the CK group; there were no significant differences in the expression levels of stu-miR482a-5p, stu-miR482b-3p, stu-miR482b-5p, and stu-miR482c between the T2 and T1 groups; Heatmap clustering revealed that the expression patterns of stu-miR482d-3p and stu-miR482d-5p were highly similar, with significantly reduced expression in the CK group and significantly increased expression in the T1 and T2 treatment groups, suggesting they may be the core miRNAs responsible for the response to treatment in root tissues.

In potato stolons (Figure 7B), compared with the CK group, the expression of all members of the miR482 family was extremely significantly increased after alkaline salt stress (T1) treatment; following EBR mediated stress relief treatment (T2), the expression of miR482 family members exhibited differential regulation: the expression levels of stu-miR482a-5p, stu-miR482b-3p, stu-miR482b-5p, and stu-miR482c were extremely significantly decreased compared to the T1 group, but remained higher than those in the CK group; The expression of stu-miR482e-3p was significantly reduced compared to the T1 group; however, there were no significant differences in the expression of stu-miR482c, stu-miR482d-3p, and stu-miR482e-5p between the T2 and T1 groups; Heatmap clustering results further confirmed that the miR482 expression profiles in the CK group differed significantly from those in the T1 and T2 groups in the stolons; stu-miR482d-5p was significantly increased in the treatment groups, suggesting it may be a key differential miRNA responding to treatment in the stolons; the overall expression levels of members such as stu-miR482a-3p, stu-miR482a-5p, and stu-miR482b-3p, among others, exhibited low overall expression levels and were only slightly increased in a few CK group samples, indicating weak specificity in their expression response.

## 3. Discussion

The miR482 family is a plant-specific small RNA that has been identified in 23 plant species to date [35], including tomato [36], soybean [37], apple [38], cotton [39], and potato [40]. Clarifying their molecular characteristics and evolutionary patterns is fundamental to understanding their functions. Based on the systematic analysis of 140 mature miR482 sequences from 26 species in this study, miR482 is most abundantly distributed in the gymnosperm European spruce, a phenomenon that is not coincidental. Previous studies have confirmed that the miR482 superfamily originated in gymnosperms and gradually diversified into distinct functional branches over the course of evolution [27].

According to the phylogenetic tree, the mature sequences of miR482 are divided into three major branches, and members of the potato stu-miR482 family are distributed across these different branches. This suggests that the evolutionary distance within miRNA families is not closely correlated with phylogenetic relationships between species [41]. Furthermore, stu-miR482a-3p, stu-miR482b-3p, and stu-miR482c, derived from the 3′ end of the precursor, cluster in clade I, while clade III consists of miRNAs derived from the 5′ end of the precursor. The mature sequences of miR482 family members 5p and 3p cluster separately, suggesting that mature sequences originating from different arms may have followed relatively independent evolutionary pathways [42].

miR482 is a widely conserved miRNA superfamily in plants. In monocotyledons, it primarily acts on long non-coding RNAs, whereas in dicotyledons, it mainly targets NBS-LRR-type disease resistance genes. This functional differentiation, coupled with sequence conservation, makes miR482 a crucial link between plant growth and development and immune regulation [27]. This study analyzed the phylogenetic relationships of pre-miR482s in potato and other species. The results showed that stu-MIR482s not only clustered with tobacco and tomato from the *Solanaceae* family but also exhibited cross-family clustering with soybean from the *Fabaceae* family and *Populus trichocarpa* from the *Salicaceae* family. This further confirms the cross-species conservation of miR482 [41]. Furthermore, secondary structure predictions revealed that all stu-pre-miR482s form a typical hairpin structure, with a minimum free energy close to the thermodynamic stability optimum of the miRNA stem-loop structure. This aligns with the typical characteristics of plant miRNA precursors, and their structural stability lays the foundation for subsequent processing into mature miRNAs and the expression of their functions [43].

The biological functions of miRNAs are ultimately realized through the regulation of target genes [44]; the prediction and analysis of target genes are critical steps in elucidating their biological functions [45]. In this study, the psRNATarget tool was used to predict potential target genes of the stu-miR482 family, encompassing categories such as transcription factors, disease resistance-related genes, and signal transduction-related genes. Among these, the identification of disease-resistance genes such as *StCC-NBS-LRR*, *StRGA3*, and *StRGA4* aligns with the known role of the miR482 family in plant pathogen immune defense, confirming the conserved function of stu-miR482 in potato disease resistance [38]; among these, NBS-LRR-type disease resistance genes are particularly noteworthy. As the largest family of plant disease resistance proteins, NBS-LRRs can recognize pathogen effectors and initiate defense responses [46]. miR482 maintains the basal expression levels of NBS-LRR mRNAs by cleaving them, a mechanism that has been reported in various plant species [47]. The stu-miR482s also target multiple transcription factors associated with salt stress responses, such as StWRKY2 and StMYB48 [48,49]. The regulation of transcription factors by miR482 suggests that it may occupy an upstream position in the regulatory network, generating a cascade effect by influencing the expression of downstream genes. Furthermore, the target genes include signaling-related genes such as *StCNGC2* and *StMAP3K1*. *StCNGC2* encodes a cyclic nucleotide-gated ion channel associated with intracellular calcium signaling [50]; *StMAP3K1* is a component of the MAPK signaling cascade [51]. In summary, the stu-miR482 family likely functions as a multifunctional regulatory hub, playing a synergistic and balancing role among immune defense, growth and development, and various signal transduction pathways.

Alkaline salt stress is one of the major abiotic stresses affecting potato growth and development, and EBR, as an important plant hormone, can enhance plant stress resistance [52,53]. In this study, stu-miR482s exhibited distinct expression patterns in roots, stems, leaves, and stolons under alkaline salt stress and EBR treatment, which may be related to differences in the sensitivity of various tissues to stress and EBR [54,55]. Of particular note is the member stu-miR482a-5p. In leaf tissue, stu-miR482a-5p exhibited low levels in the control group but was significantly upregulated following alkaline salt stress treatment; its expression declined somewhat after EBR relief but remained higher than control levels. Heatmap clustering identified it as a core differentially expressed member in the leaf response to treatment. This finding is similar to studies in mulberry, where mul-miR482a-5p was also upregulated in phloem sap following phytoplasma infection, and grafting experiments confirmed its ability to be transported from the scion to the rootstock [56]. This characteristic suggests that miR482a-5p plays a role in systemic signaling. The expression pattern in stem tissues, however, exhibits a different profile. Under alkaline salt stress, most members of the stu-miR482s family did not show significant changes; however, following EBR application, the expression levels of multiple members, including stu-miR482a-3p and stu-miR482a-5p, were significantly upregulated compared to the stress-treated group. This expression pattern may reflect the specific regulatory effect of EBR on the miR482 family. Previous studies have shown that brassinosteroids can influence miRNA transcription and processing through complex signaling networks [57,58], but the specific mechanisms remain to be further investigated.

The differences in expression were even more pronounced in the underground parts. In root tissues, the expression levels of both stu-miR482d-3p and stu-miR482d-5p were higher than those in the control group after EBR treatment, indicating a positive response to EBR treatment. In contrast, all miR482 members were extremely significantly upregulated in stolons under salt stress, which differs from other tissues. This may be because stolons are potato-specific vegetative reproductive organs and the site of tuber initiation, making them highly sensitive to stress [59]. Although the expression of stu-miR482a-5p in stolons was induced, the response was less pronounced than that of stu-miR482d-5p, further confirming the functional differentiation among family members.

In summary, stu-miR482a-5p acts as a “stress response factor” in leaves, is sensitive to EBR treatment in stems, and exhibits distinct response patterns in roots and stolons. This complex expression patterns suggests that it may be involved in signal exchange and coordination between aboveground and belowground parts. Future validation of target genes through degradome sequencing, combined with functional analysis of transgenic materials, will help further elucidate the specific role of the miR482 family in the salt tolerance mechanism of potatoes.

## 4. Materials and Methods

### 4.1. Statistical Analysis of Plant miR482 Sequences and Identification of the Potato stu-miR482 Family

A total of 140 mature miR482 sequences from 26 plant species were downloaded from miRBase (https://www.mirbase.org/; accessed on 25 February 2026), yielding 9 mature sequences and 5 precursor sequences for stu-miR482. The distribution of miR482 across these 26 plant species was visualized using Origin 2022 software.

### 4.2. Alignment Analysis and Phylogenetic Tree Construction of Plant miR482 Mature Sequences

Mature miR482 sequences from 26 plant species, including potato, tobacco, tomato, maize, grape, peach, soybean, and European spruce, were downloaded from the miRbase database and subjected to subsequent analysis. The MEGA12 software was used to perform sequence alignment analysis of miR482 and stu-miR482s sequences and to construct a phylogenetic tree of these sequences. The neighbor-joining (NJ) method was employed with 1000 bootstrap repetitions. The chiplot (https://chiplot.online/tvbot.html; accessed on 12 January 2026) was used to visualize the miR482 phylogenetic tree. 

### 4.3. Sequence Alignment, Secondary Structure Prediction, and Evolutionary Analysis of Potato stu-pre-miR482s

Sequence alignment and analysis of stu-MIR482s were performed using DNAMAN 6.0 software, while the secondary structures of stu-miR482 precursor sequences were predicted via the online RNAfold tool (http://rna.tbi.univie.ac.at/cgi-bin/RNAWebSuite/RNAfold.cgi; accessed on 20 January 2026). A phylogenetic tree was constructed for the aforementioned sequences using MEGA software based on the neighbor-joining (NJ) method, with 1000 bootstrap replicates to assess the reliability of the tree topology, and the resulting phylogenetic tree of stu-MIR482s was subsequently visualized using ChiPlot (https://chiplot.online/tvbot.html; accessed on 24 January 2026).

### 4.4. Prediction of Cis-Acting Elements in Potato stu-MIR482s

Using NCBI (https://www.ncbi.nlm.nih.gov/; accessed on 28 January 2026), we extracted the 2000 bp upstream sequence of the potato stu-MIR482s. We used the PlantCARE website (http://bioinformatics.psb.ugent.be/webtools/plantcare/html/; accessed on 19 February 2026) to predict cis-acting elements in each of these 2000 bp upstream sequences, and visualized the results using TBtools-II (Toolbox for Biologists) v2.467.

### 4.5. Prediction of the stu-MIR482s Target Gene in Potato

Predict the potential targets of miR482 with psRNATarget (http://plantgrn.noble.org/psRNATarget/; accessed on 26 February 2026) under the default parameters. In this prediction, the potato database (*Solanum tuberosum*, transcript, Group Phureja DM1-3 516R44 (CIP801092) Genome 3.4 transcript)—was adopted, and all possible target predictions were performed with the parameters of Expectation ≤ 3.5 and UPE ≤ 25. The predicted target genes were collated, and functional annotation of the candidate target genes was conducted using NCBI (https://www.ncbi.nlm.nih.gov/; accessed on 5 March 2026). Additionally, the potential regulatory relationships between stu-miR482s and their target genes were visualized with Cytoscape 3.10.0.

### 4.6. Plant Growth Conditions and Stress Treatments

The potato variety used in the experiment was ‘*Atlantic*’. The potatoes were grown in pots (light/dark cycle: 16/8 h, 23/18 °C; 4000 lx). The growing medium was thoroughly sterilized with carbendazim before sowing. The volume ratio of the growing medium was vermiculite: perlite: potting soil = 3:1:1. The growing medium depth in the pots (25 cm diameter, 15 cm height) was 13 cm. As a basal fertilizer, each pot was treated with 1.22 g/pot of urea (46% N), 1.74 g/pot of superphosphate (12% P_2_O_5_), and 1.08 g/pot of potassium sulfate (52% K_2_O). Subsequently, virus-free seed tubers were planted in the growing pots, with one tuber per container, at a planting depth of approximately 8 cm.

Our research group has previously determined that 0.1 μmol·L^−1^ EBR is most effective against alkaline salt stress [18]. Therefore, the experiment was designed with three treatments as follows: CK: normal growth conditions; T1: 200 mmol·L^−1^ NaHCO_3_; T2: 200 mmol·L^−1^ NaHCO_3_ + 0.1 μmol·L^−1^ EBR. Each treatment was arranged with three biological replicates, with six pots of seedlings per replicate. There were 18 tested seedlings per treatment and a total of 54 pots across the three treatments. Potatoes were treated with EBR 45 days after sowing. Spraying was conducted for 3 consecutive days, with EBR applied from the bottom up until a water film formed on the leaf surface. On the third day, before EBR application, NaHCO_3_ treatment was performed. For NaHCO_3_ stress, a 200 mmol·L^−1^ NaHCO_3_ solution was uniformly applied to the cultivation pots via irrigation, with 500 mL administered per pot. Three days after the third spray application, potato plant roots, stems, leaves, and stolons were collected, frozen in liquid nitrogen, and stored in an ultra-low temperature refrigerator at −80 °C for subsequent quantitative real-time PCR (qRT-PCR) analysis.

### 4.7. Analysis of the Expression Pattern of Potato stu-miR482s Under Alkaline Salt Stress and EBR Relief

Total RNA was extracted from potato samples using the SteadyPure Plant RNA Extraction Kit (Hunan Accurate Biology, Changsha, China) and stored at −80 °C for subsequent use. RNA integrity and concentration were assessed via agarose gel electrophoresis and using a Pultton P100+ ultramicro spectrophotometer (OSIC HOLDINGS, Beijing, China). RNA was reverse-transcribed using the miRNA cDNA Synthesis Kit (Kit AG11716, Hunan Accurate Biology, Changsha, Hunan, China) to synthesize first-strand cDNA. *St18S* rRNA was used as the internal reference gene [60]. Due to the short length (18–25 nt) of mature microRNAs, which precludes direct amplification by conventional PCR, the poly(A)-tailing qRT-PCR method was adopted in this study. All specific forward primers (Table 1) were synthesized by Sangon Biotech (Shanghai, China) following the specifications of this method.

qRT-PCR reactions were performed on a QuantStudio 5 system (Thermo Fisher Scientific, Waltham, MA, USA). The total reaction volume was 20 μL, consisting of 2 μL cDNA template, 0.8 μL each of forward and reverse quantitative primers, 10 μL 2X SYBR Green Pro Taq HS Premix II (Accurate Biology, Changsha, Hunan, China), and 6.4 μL ddH_2_O. The conditions of the amplification reaction were as follows: 95 °C for 30 s; 95 °C for 5 s, 60 °C for 20 s, for 40 cycles. All qRT-PCR experiments were performed with three independent biological replicates and three technical replicates. The expression levels of the 9 stu-miR482 were calculated using the 2^−ΔΔCT^ method.

### 4.8. Data Analysis

Data were compiled and calculated using Microsoft Excel and GraphPad Prism 10.0 software (GraphPad Software, Boston, MA, USA) for statistical analysis. Differences among samples were assessed using one-way analysis of variance (ANOVA) combined with Tukey multiple comparison test. Significance was defined as *p* > 0.05 (ns), *p* < 0.05 (*), *p* < 0.01 (**), and *p* < 0.001 (***). The data was represented as mean ± SD of at least three independent replicates.

## 5. Conclusions

This study systematically analyzed the genomic characteristics, target gene predictions, and expression patterns of the potato stu-miR482 family under alkaline salt stress and EBR treatment. A total of 9 mature miRNAs and 5 precursor sequences were identified, all of which form stable hairpin structures and are highly conserved among *Solanaceae* plants. Target gene prediction indicates that this family is involved in regulating various biological processes, including growth and development, disease resistance, and stress responses. Expression analysis revealed that stu-miR482s exhibit distinct tissue specificity under salt stress and EBR treatment. In leaves, stu-miR482a-5p is sensitive to salt stress and regulated by EBR; in stems, it exhibits a positive response to EBR; and expression patterns in roots and stolons also differ. Thus, stu-miR482a-5p may participate in signal communication between aboveground and belowground parts and play a key role in regulating salt tolerance in potatoes.

## Figures and Tables

**Table 1:** Genes and Primer Sequences for the Genes Used for RT-qPCR Verification.

Gene ID	Forward Primer
*St1*8s RNA	TTAGAGGAAGGAGAAGTCGTAACAA
Stu-miR482a-3p	TTTCCAATTCCACCCATTCCTA
Stu-miR482a-5p	GGAATTGGTGGATTGGAAAGC
stu-miR482b-3p	TTACCGATTCCCCCCATTCCAA
stu-miR482b-5p	GGAGTGGGTGGCATGGTAAGA
stu-miR482c	TTTCCTATTCCACCCATGCCAA
stu-miR482d-3p	TCTTGCCTACACCGCCCATGCC
stu-miR482d-5p	CGTGAGTGGTGGGGTAAGATA
stu-miR482e-3p	TCTTGCCAATACCGCCCATTCC
stu-miR482e-5p	AGTGGGTGGTGTGGTAAGATT

Note: Only forward primers are provided for mature miRNAs.

## Data Availability

The datasets used and analyzed in the current study are available from the corresponding author on reasonable request.

## Abbreviations

The following abbreviations are used in this manuscript:

NJ	Neighbor-Joining
qRT-PCR	quantitative real-time PCR
EBR	24-epibrassinolide
